# School-Located Influenza Vaccination Reduces Community Risk for Influenza and Influenza-Like Illness Emergency Care Visits

**DOI:** 10.1371/journal.pone.0114479

**Published:** 2014-12-09

**Authors:** Cuc H. Tran, Jonathan D. Sugimoto, Juliet R. C. Pulliam, Kathleen A. Ryan, Paul D. Myers, Joan B. Castleman, Randell Doty, Jackie Johnson, Jim Stringfellow, Nadia Kovacevich, Joe Brew, Lai Ling Cheung, Brad Caron, Gloria Lipori, Christopher A. Harle, Charles Alexander, Yang Yang, Ira M. Longini, M. Elizabeth Halloran, J. Glenn Morris, Parker A. Small

**Affiliations:** 1 Department of Environmental and Global Health, College of Public Health and Health Professions, University of Florida, Gainesville, Florida, United States of America; 2 Emerging Pathogens Institute, University of Florida, Gainesville, Florida, United States of America; 3 Clinical Translational Science Institute, University of Florida, Gainesville, Florida, United States of America; 4 Department of Epidemiology, College of Public Health and Health Professions, University of Florida, Gainesville, Florida, United States of America; 5 Department of Epidemiology, College of Medicine, University of Florida, Gainesville, Florida, United States of America; 6 Vaccine and Infectious Disease Division, Fred Hutchinson Cancer Research Center, Seattle, Washington, United States of America; 7 Department of Biology, College of Liberal Arts and Sciences, University of Florida, Gainesville, Florida, United States of America; 8 Fogarty International Center, National Institutes of Health, Bethesda, Maryland, United States of America; 9 Department of Pediatrics, College of Medicine, University of Florida, Gainesville, Florida, United States of America; 10 Florida Department of Health in Alachua County, Gainesville, Florida, United States of America; 11 College of Nursing, University of Florida, Gainesville, Florida, United States of America; 12 College of Pharmacy, University of Florida, Gainesville, Florida, United States of America; 13 Alachua County Public Schools, Gainesville, Florida, United States of America; 14 Partnership for Strong Families, Gainesville, Florida, United States of America; 15 University of Florida Health Integrated Data Repository, UF Health, Gainesville, Florida, United States of America; 16 Department of Health Services Research, Management and Policy, College of Public Health and Health Professions, University of Florida, Gainesville, Florida, United States of America; 17 Florida Department of Health, Tallahassee, Florida, United States of America; 18 Department of Biostatistics, College of Public Health and Health Professions, University of Florida, Gainesville, Florida, United States of America; 19 Department of Biostatistics, Colleges of Medicine, University of Florida, Gainesville, Florida, United States of America; 20 Department of Biostatistics, University of Washington, Seattle, Washington, United States of America; 21 Department of Medicine, College of Medicine, University of Florida, Gainesville, Florida, United States of America; 22 Department of Pathology, College of Medicine, University of Florida, Gainesville, Florida, United States of America; University of Hong Kong, Hong Kong

## Abstract

**Background:**

School-located influenza vaccination (SLIV) programs can substantially enhance the sub-optimal coverage achieved under existing delivery strategies. Randomized SLIV trials have shown these programs reduce laboratory-confirmed influenza among both vaccinated and unvaccinated children. This work explores the effectiveness of a SLIV program in reducing the community risk of influenza and influenza-like illness (ILI) associated emergency care visits.

**Methods:**

For the 2011/12 and 2012/13 influenza seasons, we estimated age-group specific attack rates (AR) for ILI from routine surveillance and census data. Age-group specific SLIV program effectiveness was estimated as one minus the AR ratio for Alachua County versus two comparison regions: the 12 county region surrounding Alachua County, and all non-Alachua counties in Florida.

**Results:**

Vaccination of ∼50% of 5–17 year-olds in Alachua reduced their risk of ILI-associated visits, compared to the rest of Florida, by 79% (95% confidence interval: 70, 85) in 2011/12 and 71% (63, 77) in 2012/13. The greatest indirect effectiveness was observed among 0–4 year-olds, reducing AR by 89% (84, 93) in 2011/12 and 84% (79, 88) in 2012/13. Among all non-school age residents, the estimated indirect effectiveness was 60% (54, 65) and 36% (31, 41) for 2011/12 and 2012/13. The overall effectiveness among all age-groups was 65% (61, 70) and 46% (42, 50) for 2011/12 and 2012/13.

**Conclusion:**

Wider implementation of SLIV programs can significantly reduce the influenza-associated public health burden in communities.

## Introduction

Influenza is an important vaccine-preventable disease. Children demonstrate the highest levels of influenza transmission, [1]–[3] suggesting that reducing the rate of infection in this population could indirectly lead to substantial risk reduction among other community members. [4] Vaccination of schoolchildren, through school-located influenza vaccination (SLIV) programs promises to be an efficient complementary strategy for increasing the sub-optimal influenza immunization rates achieved through existing immunization delivery programs. [5] Recently, the United Kingdom announced a £100 million expansion of their influenza vaccination program to include healthy schoolchildren [6] and began to pilot these SLIV programs this past season [7].

Three decades of computer modeling [8], [9] and epidemiologic studies [10] support the concept that immunizing children can indirectly protect unimmunized adults in the same community. Mathematical modeling suggests that vaccination of 20% of children 5–18 years could be more effective at reducing influenza-associated mortality among adults older than 64 years than immunizing 90% of the latter age-group. [11] Furthermore, immunizing 70% of schoolchildren could prevent outbreaks from occurring in a community. [11] Field studies show that schools are virus exchange systems, [12] and children shed greater quantities of virus and for longer periods of time than adults. A retrospective study of the first nationwide influenza vaccination program suggested that one life was saved for every 420 Japanese schoolchildren immunized. [10] More recently, a study in California reported a 30% reduction in laboratory-confirmed infection among the students enrolled in schools with an SLIV program, relative to schools lacking such a program [13].

The level of indirect effectiveness predicted by mathematical modeling investigations [9], [11], [14] and intervention trials [15]–[17] has yet to be corroborated through empirical assessment of routine SLIV programs delivering live attenuated influenza vaccine (LAIV) to schoolchildren of all ages. This report estimates the effectiveness of an ongoing “opt in” SLIV program implemented in all preK-12 schools (public and private) of Alachua County, Florida, USA for the prevention of influenza-like illness (ILI) outpatient visits at emergency departments and urgent care centers.

## Methods

### Influenza Immunization in the United States

#### Non-Alachua Counties

In Florida (and elsewhere in the USA), the majority of routinely-administered vaccinations are funded through private health insurance. Children on Medicaid (a government-sponsored health care program for low income families) or who have no insurance are eligible to receive free vaccine through the Vaccines for Children Program (VFC) paid for by the US federal government. VFC recipients are required to obtain their vaccine at a local health department or specific VFC providers. Regardless of insurance status, most children who receive influenza vaccination do so at a health care facility.[18] Based on a recent analysis of confirmed seasonal influenza vaccinations, we expect the vaccination coverage among privately insured children 5–8 years and 9–17 years to be between 20%–31% and 9%–18% respectively [19].

#### Alachua County

Implementation of the Alachua County SLIV program is described in detail elsewhere. [5] In brief: the county health department, the public school system, the local pediatricians, the University of Florida, and the county government support this major community initiative to control influenza. The program's goal is to protect the community from influenza by eliminating the financial impediments and access-to-care barriers that tend to prevent schoolchildren from receiving influenza vaccine. The pilot phase of the “opt in” SLIV program began with MedImmune providing free LAIV to kindergarten through 8^th^ grade students in November 2006. The comprehensive program was launched at the start of the 2009/10 school year with the support of local pediatricians and 29 other community partners. Vaccine was made available through the Florida Department of Health and programmatic support was funded through a county health sales surtax, the Children's Miracle Network, and the AvMed Health Plans. In 2010/11, the program was expanded to include high school students. Children ineligible to be vaccinated with LAIV (mostly children with asthma) at school are referred to local pediatricians or other healthcare providers for vaccination with inactivated influenza vaccine.

### Data Sources and Analysis

#### Data sources

Influenza vaccination rates for Alachua County were obtained from the Florida Department of Health's Florida SHOTS Vaccine Registry. Through collaboration between the Health Department and community pediatricians, all influenza vaccinations of Alachua County residents were entered into the Florida SHOTS Vaccine Registry. Because the state registry does not require all medical providers to report influenza vaccination, comparable vaccination coverage data were not available for other counties. However, we expect the vaccination coverage to be between 18%–31% among privately insured schoolchildren between 5–17 years old [19].

Weekly influenza and influenza-like illness (ILI) associated outpatient visits to emergency departments and urgent care centers were obtained from Florida's Electronic Surveillance System for the Early Notification of Community-based Epidemics (ESSENCE). Emergency department and urgent care centers will be referred to as emergency care (EC). This system systematically downloads chief complaint data on a daily basis for all participating EC facilities within Florida. For the 2011/12 and 2012/13 school years, ESSENCE reporting covered approximately 79% of eligible facilities statewide, 65% in Region 3 (12 northeastern counties of the state, excluding Alachua County), and 100% in Alachua County (intervention); levels of participation by EC facilities before 2011 were not felt to be adequate to permit inclusion of ESSENCE data prior to 2011 in this study. The ESSENCE case definition for influenza and influenza like illness (ILI) is an outpatient visit to a reporting facility with a chief complaint of “influenza”, “flu”, or “fever” plus “cough” and/or “sore throat”. Investigators received the number of ILI-associated weekly visits aggregated by the patient's permanent county of residence and age-group (0–4, 5–17, 18–44, 45–64, and 65 years and older). County and age-group specific resident counts were obtained from the 2010 US Census, [20] and school enrollment data was obtained from the Florida Department of Education [21]. All residents of a county were considered to be at-risk for ILI-associated EC visits.

**Figure 1:**
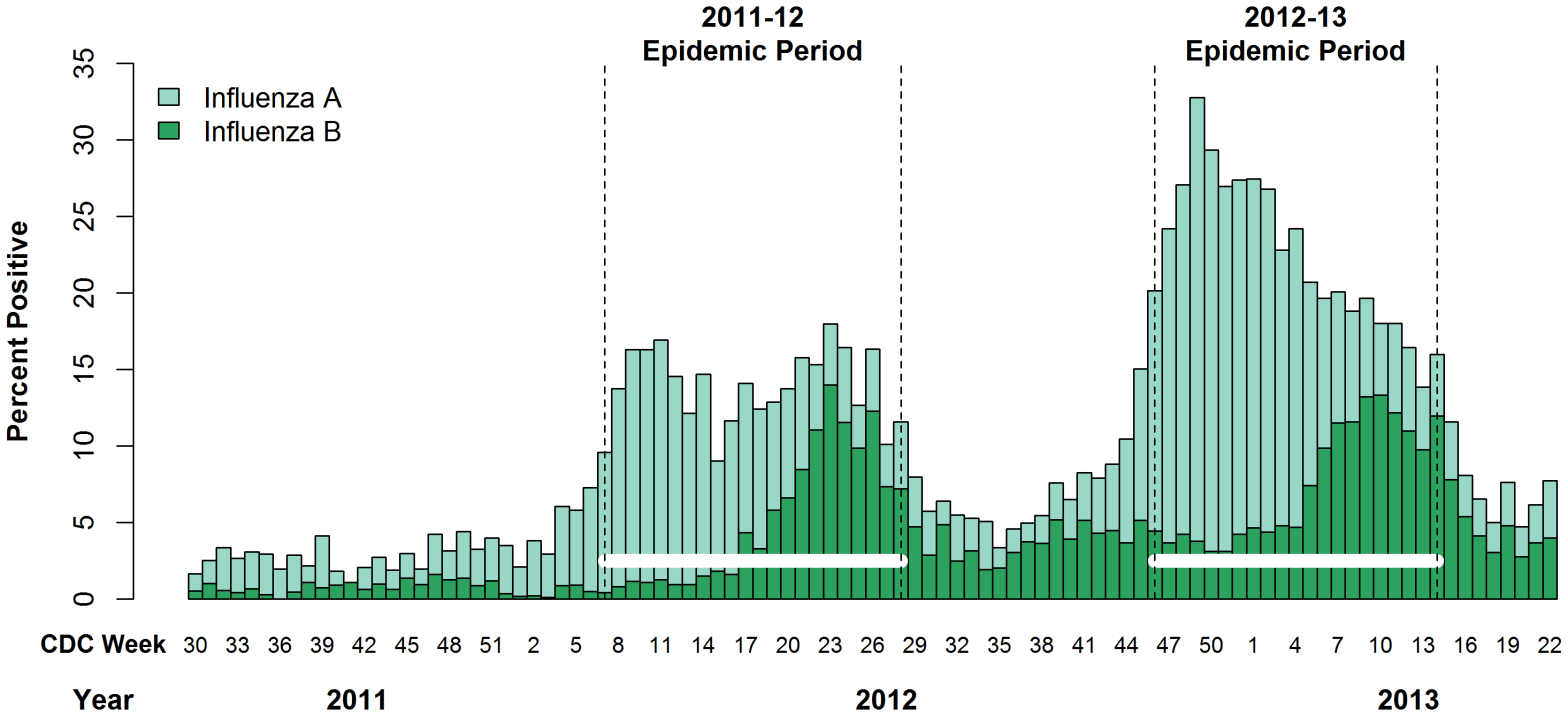
Establishing Influenza Epidemic Periods. The proportion of the laboratory specimens positive for influenza A (light bar) and B (dark bar) viruses among isolates submitted by the states of the Health and Human Services Southeastern Health Region 4 to the National Respiratory and Enteric Virus Surveillance System maintained by the United States Centers for Disease Control and Prevention (CDC). Data are shown for 2011/12 and 2012/13. [24] Influenza epidemic periods (horizontal bars) for each year are defined using CDC criteria [22], [23].

To reduce the potential that ILI will be misclassified as being caused by influenza infection (*i.e.*, false positive), only ESSENCE surveillance data collected during influenza epidemic periods were analyzed. Influenza epidemic periods were defined for the 2011/12 and 2012/13 school years using the Centers for Disease Control and Prevention (CDC) guidelines [22], [23] and laboratory-based surveillance data for the Southeastern Health and Human Services Region 4 of the United States (Fig. 1). [24] Specifically, an influenza epidemic period begins with the first set of two or more consecutive weeks during which the proportion of respiratory isolates testing positive for influenza A or B exceeds 10% and remains elevated during the subsequent weeks. The end of the influenza season is defined as the first of a period of two or more consecutive weeks during which each week accounts for <2% of the total number of specimens that tested positive for influenza during the entire season. Data from sentinel providers and viral surveillance were not utilized because this information is not systematically collected across Florida.

#### Statistical methods

The attack rates (AR) were estimated as the number of cases of ILI-associated emergency care visits per 100,000 resident population. ARs were estimated by epidemic period and age-group, for each of the following geographic regions: Alachua County; the 12 surrounding counties (Region 3); and all non-Alachua counties in Florida. The SLIV program effectiveness at reducing the risk of ILI-associated EC outpatient visits was estimated for each combination of epidemic period and age-group as one minus the ratio of the corresponding ARs for Alachua County and a comparison region (either Region 3 or Florida). The effectiveness among 5–17 year-olds is considered to estimate the overall protection, both direct and indirect, associated with the Alachua County SLIV program. [25] Effectiveness for all other age-groups is considered to estimate the age-specific level of indirect protection associated with the Alachua County SLIV program. [25] Data analysis was conducted using the *epiR* package in R v3.0.3. [26], [27] Ninety-five percent confidence intervals (CI) for the AR and SLIV program effectiveness estimates are respectively based upon exact and asymptotic methods for estimating the standard error.

#### Sensitivity analysis for possible observation bias in SLIV effectiveness estimates

A sensitivity analysis was conducted to investigate potential bias due to differential levels of ascertainment of ILI-associated outpatient emergency care visits between Alachua County and the comparison areas. Since the number of ILI outpatient EC cases reported by ESSENCE facilities may be a function of the overall EC visit volume, the ARs for ILI-associated outpatient EC visits were standardized to the overall visit volume seen in Alachua County ESSENCE facilities. The existence of the SLIV program in Alachua County could plausibly exert influences on the sensitivity and/or the specificity of the county's ESSENCE system for ILI-associated outpatient EC visits, potentially through elevated vigilance by those responsible for reporting to ESSENCE. Any added vigilance among the surveillance staff at Alachua County ESSENCE facilities (relative to reporting ECs in the comparison areas) should be at least partially apparent in the rates of other chief complaints recorded by ESSENCE, even for chief complaints that are reasonably expected to be weakly associated or unassociated with the direct and/or indirect effects of the LAIV delivered by the SLIV program. The impact of this potential source of differential ascertainment was investigated by adjusting the point estimate for the SLIV program's effectiveness against ILI-associated outpatient EC visits using the estimate of the program's effect on each of three negative control outcomes (outpatient EC visits for gastrointestinal illness, respiratory illness other than ILI, or physical injury), as well as the combined effect on all three. A detailed description of the analytic approach for the sensitivity analysis is provided in S1 Text.

#### Ethics Statement

This research was reviewed and approved by the University of Florida Institutional Review Board. The aggregated data was anonymized and de-identified prior to analysis; therefore, informed consent was not required.

## Results

### Influenza vaccination in Alachua County

During the 2011/12 influenza season, approximately 47% of school-age (5–17 years) residents of Alachua County received influenza vaccination, with 10,490 students vaccinated with LAIV through the SLIV program and an additional 1,936 students receiving any type of vaccine from a non-SLIV source (Table 1). The overall influenza vaccination coverage rate among school-age children for the 2012/13 season was approximately 50%, with 11,188 students vaccinated through the SLIV program and an additional 2,391 students receiving any type of influenza vaccine from other sources. In both years, a small subset of schoolchildren received their LAIV at their medical provider instead of through the SLIV program.

**Table 1:** Influenza vaccination coverage (%) among children under-18 years of age for Alachua County.

Age-Group	Vaccination Rate	Alachua County^a^^,^ b							
		School Year	2006/07	2007/08	2008/09	2009/10	2010/11	2011/12	2012/13
**0–4 Years**	**Overall**		-	-	-	**12%**	**16%**	**16%**	**16%**
**5–10 Years**	**Overall Elementary**		**>27%**	-	-	**67%**	**67%**	**63%**	**65%**
	SLIV Program		27%	-	-	60%	59%	54%	55%
**11–13 Years**	**Overall Middle**		**>24%**	-	-	**43%**	**41%**	**43%**	**49%**
	SLIV Program		24%	-	-	40%	36%	36%	40%
**14–17 Years**	**Overall High**		-	-	-	**6%**	**23%**	**24%**	**30%**
	SLIV Program		-	-	-	-	18%	19%	22%
**5–17 Years**	**Overall School-aged**		**>25%**	-	-	**42%**	**48%**	**47%**	**50%**

The overall vaccination coverage levels for each age group includes all children vaccinated with at least one dose of the live attenuated influenza vaccine (LAIV) or inactivated influenza vaccine (IIV) received from the any provider. The SLIV program vaccination coverage level includes a subset of children vaccinated with LAIV through that program's activities. Vaccination coverage levels were based upon information recorded in the Florida SHOTS Vaccine Registry.

a Denominator based on 2010 Census Data [16] for the 0–4 age-group. Vaccination rates for ages two years and younger were not available.

b Denominators based on student enrolment [17] by school year for each school type.

### Influenza Epidemic Period

The mild 2011/12 epidemic period [22] was defined as being 20-weeks long, extending from CDC week 7 through week 26 of 2012. The moderately-severe 2012/2013 epidemic period [23] was defined as being 21-weeks long, extending from CDC week 44 of 2012 to week 13 of 2013. The CDC Region 4 definition for the 2011/12 epidemic period was consistent with laboratory-based surveillance data collected by the Florida Department of Health, but the 2012/13 epidemic period saw transmission of influenza beginning earlier in Florida than the regional data. [28] Formulations of both LAIV and the inactivated influenza vaccine delivered in the US during the 2011/12 and 2012/13 school years were considered to be homologous to the strains circulating during the subsequent epidemic periods [22], [23], [29].

### Cases of emergency department and urgent care ILI visits

**Figure 2:**
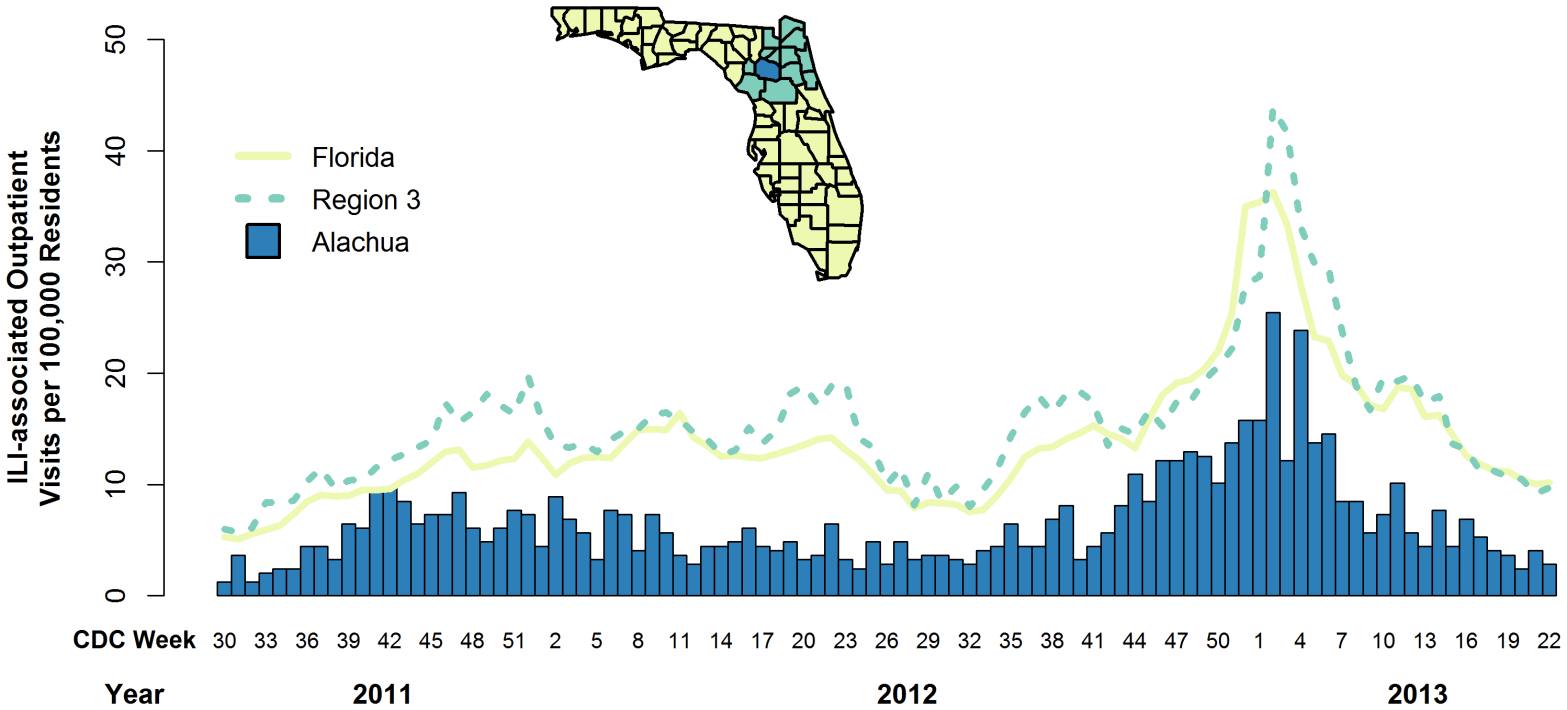
Number of cases (per 100,000 residents) of influenza-like illness (ILI) associated outpatient visits by geographic region and calendar week. Chief complaint information is reported for outpatient visits to 183 emergency departments and urgent care facilities located throughout the state of Florida. Rates are presented for Alachua County (bars), the location of a novel school-located influenza vaccination program, and two comparison regions: the nearby 12 counties (Region 3, dashed line) and all non-Alachua counties (Florida, solid line). The map inset depicts the locations of Alachua and Region 3 within the state of Florida.

The weekly rate of ILI-associated outpatient EC visits per 100,000 population were consistently higher in the comparison regions (Region 3 and Florida) than in Alachua County (Fig. 2). The same trend was observed for age-group specific ARs (Table 2), though the relative magnitude of the difference between Alachua County and the comparison regions dropped with increasing age.

**Table 2:** The attack rates (95% confidence limits) per 100,000 residents for cases of influenza-like illness associated outpatient visits to sentinel emergency departments and urgent care facilities, influenza epidemic season, geographic region, and age-group.

		SLIV Program Area		Comparison Areas			
Age-Group		Alachua County		All Non-Alachua Counties		Region 3	
	Epidemic Period	2011/12	2012/13	2011/12	2012/13	2011/12	2012/13
**0–4 Years**		**168**	**344**	**1,550**	**2,207**	**1,606**	**2,099**
		(98, 239)	(244, 445)	(1,526, 1,573)	(2,179, 2,235)	(1,535, 1,677)	(2,018, 2,180)
**5–17 Years**		**99**	**199**	**474**	**680**	**559**	**652**
		(64, 134)	(149, 248)	(466, 482)	(670, 689)	(533, 585)	(624, 680)
**18–44 Years**		**109**	**307**	**225**	**438**	**272**	**507**
		(91, 128)	(276, 339)	(222, 229)	(433, 443)	(259, 284)	(490, 524)
**45–64 Years**		**62**	**205**	**89**	**227**	**83**	**238**
		(41, 82)	(168, 242)	(86, 91)	(223, 232)	(75, 90)	(225, 251)
**≧65 Years**		**64**	**199**	**68**	**227**	**70**	**193**
		(34, 94)	(146, 253)	(66, 71)	(222, 232)	(61, 80)	(177, 208)

### Effectiveness

**Figure 3:**
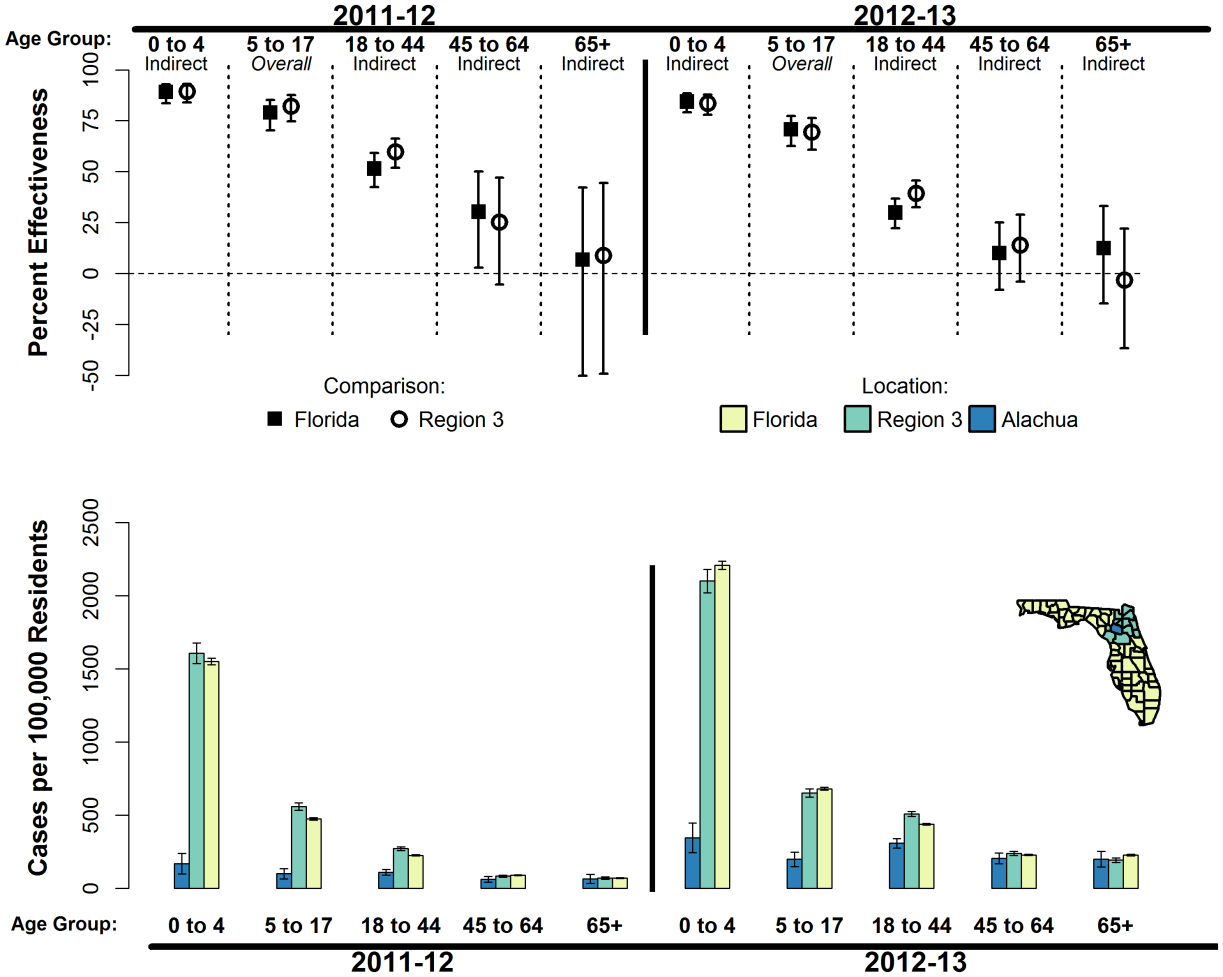
Estimated effectiveness of the Alachua County school-located influenza vaccination (SLIV) program (upper panel) and attack rates for influenza-like illness (ILI) associated outpatient visit to sentinel emergency care facilities (lower panel) by age-group and epidemic periods. School-age children (5–17 years) are the target age-group for the SLIV; thus, the SLIV effect in this age-group is considered a measure of the program's overall effectiveness. SLIV effects in all other age-groups are considered measures of indirect effectiveness. SLIV effectiveness is defined as 1 minus the ratio of the age-group specific seasonal attack rates for ILI-associated outpatient visits in Alachua County versus the rates for one of two comparison regions: the surrounding 12 counties (Region 3, open circles) and all non-Alachua counties of Florida (squares). Vertical error bars represent 95% confidence intervals.

Due to the relative similarity between the age-group specific ARs for the Region 3 and Florida comparison regions (Table 2), only effectiveness estimates comparing Alachua County and Florida are presented in the main text, but estimates involving Region 3 were similar in value (Fig. 3, Table 3, and Table 4). The estimated overall effectiveness of the Alachua SLIV program among 5–17 year-olds was 79% (95% CI: 70%, 85%) for the milder 2011/12 epidemic period and 71% (95% CI: 63%, 77%) for the moderately-severe 2012/13 period (Fig. 3, Table 3, and Table 4). Among all non-school age residents, *i.e.*, excluding 5–17 year-olds, the estimated indirect effectiveness was 60% (95% CI: 54%, 65%) for 2011/12 and 36% (95% CI: 31%, 41%) for 2012/13. The overall effectiveness among all age-groups was 65% (95% CI: 61%, 70%) for 2011/12 and 46% (95% CI: 42%, 50%) for 2012/13.

**Table 3:** Unadjusted and bias-corrected effectiveness estimates of the Alachua County school-located influenza vaccination program for 2011/12 epidemic period.

Basis for Correction	All Non-Alachua Counties of Florida						Region 3					
	0–4 Years	5–17 Years	18–44 Years	45–6 Years	≥65 Years	All Ages	0–4 Years	5–17 Years	18–44 Years	45–64 Years	≥65 Years	All Ages
**Unadjusted (Primary)**	**89.1**	**79.0**	**51.5**	**30.3**	**6.7**	**65.4**	**89.5**	**82.2**	**59.8**	**25.2**	**8.9**	**70.0**
	83.5, 92.8	70.2, 85.3	42.4, 59.1	2.8, 50.0	−50.3, 42.1	60.6, 69.5	84.0, 93.1	74.7, 87.5	52.0, 66.3	−5.4, 47.0	−49.2, 44.4	65.8, 73.6
**EC Visit Volume**	**89.0**	**79.6**	**44.2**	**44.6**	**33.0**	**65.6**	**86.8**	**78.3**	**38.9**	**25.6**	**17.2**	**61.8**
	84.6, 94.0	72.5,87.3	35.0, 53.3	23.9, 61.9	−3.5, 62.3	61.3, 70.6	81.8, 92.9	70.1, 86.4	27.0, 48.1	−1.2, 48.9	−23.8, 54.6	57.4, 66.9
**Other Resp. Illness**	**80.7**	**68.4**	**27.4**	**33.0**	**16.0**	**50.9**	**78.2**	**68.4**	**26.2**	**12.8**	**−7.0**	**47.7**
	72.7, 89.2	57.4, 79.6	15.2, 40.3	5.8, 53.9	−27.5, 52.1	44.8, 58.0	68.8, 87.9	55.7, 79.5	11.6, 39.2	−22.5, 40.6	−59.1, 39.4	40.6, 54.6
**GI Illness**	**84.1**	**77.0**	**40.3**	**40.9**	**21.2**	**62.5**	**84.8**	**78.5**	**34.4**	**20.8**	**2.0**	**59.8**
	77.2, 91.1	67.8, 85.4	29.5, 49.7	19.3, 59.5	−19.5, 58.2	57.9, 67.5	77.9, 91.5	69.7, 86.4	21.2, 44.5	−7.2, 46.1	−49.4, 48.4	54.7, 65.0
**Injury**	**89.8**	**79.0**	**34.7**	**33.6**	**26.6**	**59.6**	**89.2**	**79.8**	**38.9**	**26.7**	**22.5**	**62.0**
	85.6, 94.5	70.1, 86.9	23.5, 45.4	11.0, 55.1	−13.4, 58.5	54.4, 65.3	84.7, 94.2	71.2, 87.0	27.7, 48.9	−1.1, 51.2	−18.7, 56.9	57.1, 67.3
**All**	**85.4**	**75.2**	**34.4**	**36.0**	**21.4**	**57.9**	**84.7**	**76.1**	**33.4**	**20.3**	**6.7**	**56.9**
	79.6, 91.9	65.6, 84.1	23.2, 45.0	12.4, 56.2	−20.5, 56.4	52.7, 63.8	78.4, 91.6	66.6, 84.8	20.4, 43.6	−8.8, 45.3	−40.4, 48.6	51.4, 62.7

Estimates of the effectiveness (%, bold font) of program toward reducing the number of cases for influenza-like illness associated outpatient visits to sentinel emergency departments and urgent care facilities (ECs), by comparison area, age-group, and correction factors. Ninety-five percent confidence limits are presented directly below each effectiveness estimate. With the exception of the unadjusted effectiveness, all estimates are standardized for differences between Alachua County and the comparison regions in the overall volume of outpatient EC visits (see text of S1 Text for details). Bias correction is also conducted based upon the effects of the SLIV program on the attack rates for outpatient EC visits for three negative control chief complaints (other respiratory illness, gastrointestinal illness, and physical injury), plus the geometric mean of the three effects (All).

**Table 4:** Unadjusted and bias-corrected effectiveness estimates of the Alachua County school-located influenza vaccination program for 2012/13 epidemic period.

Basis for Correction	All Non-Alachua Counties of Florida						Region 3					
	0–4 Years	5–17 Years	18–44 Years	45–64 Years	≥65 Years	All Ages	0–4 Years	5–17 Years	18–44 Years	45–64 Years	≥65 Years	All Ages
**Unadjusted (Primary)**	**84.4**	**70.8**	**29.9**	**9.9**	**12.3**	**46.1**	**83.6**	**69.5**	**39.4**	**13.9**	**−3.2**	**48.5**
	79.1, 88.3	62.5, 77.2	22.3, 36.7	−8.1, 25.0	−14.8, 33.1	41.8, 50.1	78.0, 87.8	60.8, 76.3	32.6, 45.6	−4.1, 28.8	−36.7, 22.1	44.2, 52.5
**EC Visit Volume**	**85.2**	**71.4**	**20.0**	**26.8**	**32.7**	**47.9**	**79.7**	**62.6**	**10.2**	**13.9**	**5.6**	**36.1**
	81.0, 89.2	64.1, 78.1	12.6, 27.9	11.8, 40.5	15.7, 49.2	42.7, 51.8	74.2, 85.3	53.0, 71.2	1.0, 18.9	−3.2, 32.1	−21.5, 29.8	30.5, 40.8
**Other Resp. Illness**	**76.5**	**58.1**	**−14.5**	**5.6**	**12.9**	**22.3**	**69.2**	**48.4**	**−22.8**	**−8.4**	**−25.1**	**7.3**
	70.4, 83.3	46.3, 69.1	−27.6, −1.1	−14.2, 24.9	−9.4, 35.8	14.4, 28.2	61.6, 77.7	33.3, 61.7	−37.9, −7.8	−31.2, 14.8	−59.8, 7.6	−0.6, 14.3
**GI Illness**	**75.9**	**65.0**	**14.7**	**21.1**	**25.5**	**41.6**	**73.8**	**61.5**	**6.0**	**9.4**	−**1.6**	**32.9**
	69.0, 82.5	55.2, 73.5	6.9, 23.8	4.8, 37.3	5.1, 45.0	35.4, 45.9	66.3, 81.3	51.3, 70.5	−3.6, 15.5	−8.7, 28.2	−33.4, 26.6	26.0, 37.6
**Injury**	**85.4**	**69.6**	**5.5**	**14.6**	**34.5**	**38.8**	**83.4**	**66.0**	**11.7**	**19.2**	**22.2**	**38.4**
	81.1, 89.6	60.4, 77.1	−5.2, 15.0	−3.7, 32.9	14.4, 50.2	32.0, 43.7	78.9, 88.1	56.7, 74.7	−0.5, 20.8	0.7, 36.3	−1.9, 41.3	31.9, 43.9
**All**	**79.8**	**64.5**	**2.7**	**14.0**	**24.8**	**34.8**	**76.3**	**59.3**	**−0.7**	**7.4**	**0.4**	**27.4**
	74.5, 85.3	54.9, 72.8	−7.1, 12.3	−3.4, 30.8	4.3, 44.0	28.3, 39.7	70.3, 82.9	48.1, 69.1	−11.6, 9.7	−11.6, 27.0	−29.1, 26.2	20.6, 32.6

Estimates of the effectiveness (%, bold font) of program toward reducing the number of cases for influenza-like illness associated outpatient visits to sentinel emergency departments and urgent care facilities (ECs), by comparison area, age-group, and correction factors. Ninety-five percent confidence limits are presented directly below each effectiveness estimate. With the exception of the unadjusted effectiveness, all estimates are standardized for differences between Alachua County and the comparison regions in the overall volume of outpatient EC visits (see text of S1 Text for details). Bias correction is also conducted based upon the effects of the SLIV program on the attack rates for outpatient EC visits for three negative control chief complaints (other respiratory illness, gastrointestinal illness, and physical injury), plus the geometric mean of the three effects (All).

For both epidemic periods, indirect protection associated with the Alachua SLIV program decreased with increasing age (Fig. 3, Table 3, and Table 4), excluding 5–17 year-olds. During the mild 2011/12 period, the SLIV program was associated with an indirect protection of 89% (95% CI: 84%, 93%) among 0–4 year-olds, 52% (95% CI: 42%, 59%) among 18–44 year-olds, 30% (95% CI: 3%, 50%) among 45–64 year-olds, and 7% (95% CI: −50%, 42%) among those 65 years and older (Fig. 3, Table 3, and Table 4). For the moderately-severe 2012/13 epidemic period, the estimated indirect protection was 84% (95% CI: 79%, 88%) among 0–4 year-olds, 30% (95% CI: 22%, 37%) among 18–44 year-olds, 10% (95% CI: −8%, 25%) among 45–64 year-olds, and 12% (95% CI: −15%, 33%) among those 65 and older. The CIs for the indirect effectiveness estimates among those 65 years and older included the null effect of 0%.

As reflected in Fig. 2, and S1 and S2 Figures, decreases in the rate of ILI-associated outpatient EC visits in Alachua County were noted during both epidemic and non-epidemic periods, particularly among children. Because of concerns that this might be associated with an underlying ascertainment bias, we used data on total visits and visits for other clinical syndromes to correct our estimates of vaccine effectiveness. We did find variability in overall rates of EC utilization among counties, with Alachua County residents in the 18–44 year old age-group being less likely to go to the EC, and those over 44 being slightly more likely to visit the EC than persons in the comparison counties. However, as shown in Table 3 and Table 4, when we corrected the estimates of vaccine effectiveness for rates of EC visits and for differences in rates of presentation with other diagnostic categories (gastrointestinal illness, respiratory illness other than ILI, and physical injury), the reductions in the observed effectiveness for the 0–4 and 5–17 year old age-groups were relatively minor; we did see a fairly substantial drop in effectiveness in the 18–44 year age-group, but then increases in vaccine effectiveness estimates in older age-groups.

## Discussion

During the 2011/12 and 2012/13 influenza epidemic period, a substantial reduction in the risk of ILI-associated outpatient EC visits among under 65 year-old residents of Alachua County was associated with vaccination of approximately 50% of all school-age children residing within the county through a routine school-located influenza vaccination program. The SLIV program is associated with a 70–79% reduction in the risk of medically-attended ILI among the 5–17 year-olds, a group with an overall vaccination coverage of 47–51%. The risk of ILI-associated outpatient EC visits among 0–4 year-old Alachua County residents was 84–89% lower than the rest of Florida, providing strong evidence that vaccination of school-aged children contributes indirect protection to younger members of the community.

We found some evidence of an indirect effect of the SLIV program among working-age adults (18–44 years), although the effect was relatively small after correction for overall EC visits volumes and/or the rates of presentation to reporting ECs for other negative control chief complaints. We would note, however, that the population in this age-group in Alachua County is highly atypical for Florida counties, including, as it does, approximately 50,000 students attending the University of Florida. These students constitute some 28% of the total population of the County, and have an immunization rate of <10%. Without this admixture of University students, who do not fit into the typical model of adults living at home with children, we wonder if the indirect vaccine effectiveness for the 18–44 year age-group may have been higher. The level of indirect effectiveness did continue to drop as we moved into older age-groups. This trend of decreased levels of indirect protection with increased age is consistent with higher vaccination coverage rates among older adults, [30], [31] as well as with higher levels of full or partial acquired immunity associated with a longer history of exposure to vaccination and/or natural infection. Across all age-groups, the effectiveness of the SLIV program was found to be greater in the mild season (2011/2012), which is consistent with the theoretical prediction that indirect vaccine effects are greater in low transmission intensity settings [9].

Similar to other studies, [32] we did not observe the same level of indirect effectiveness among the elderly as was reported for the previously noted 1977–87 study of mandatory influenza immunization in Japan [10] and suggested by mathematical modeling studies. [8], [9] One possible explanation is the consistently high influenza vaccination coverage rates among the elderly in the US [30], [31] that may leave little room for an indirect effect of the SLIV program. Furthermore, evidence suggests that the contact rate between schoolchildren and the elderly is substantially lower in the US relative to Japan, reducing the risk of exposure and the potential for indirect protection. A US Census report estimated that ∼5% of families live in multigenerational households, [33] much lower than in Japan [10].

Despite the ecologic nature of this study and its reliance on data collected by a surveillance system for clinical disease, the results of this study are consistent with smaller scale randomized trials and community-based studies. Our results among the 5–17 age-group are consistent with a study using PCR-confirmed influenza as the outcome, which saw a 60% reduction in risk of influenza in an elementary school with 50% of their students vaccinated with LAIV. [13] A county-wide program with a 41–48% LAIV vaccination rate reported 35% fewer emergency department visits based on ICD-9 ILI among the target age-group. [34] A similar study in Maryland observed fewer cases among the 5–11 and 19–64 year-old residents of communities with SLIV programs, relative to those lacking this type of public health intervention strategy. [32] Our analytic approach has the advantage of harnessing existing surveillance systems that systematically collect clinical data throughout the State, overcoming the difficulty of obtaining parental consent to collect clinical specimens from children for laboratory confirmation [35].

It is of interest that reductions in ILI rates in Alachua County were seen during both “epidemic” and “non-epidemic” time periods. Our initial concern was that this observation could be reflective of ascertainment or other bias in reporting of Alachua County cases through the ESSENCE system. However, despite an extensive sensitivity analysis we could not demonstrate the presence of any significant biases in relative levels of the ascertainment of ILI-associated outpatient EC visits for Alachua County versus the comparison regions, nor did we see any major biases in vaccine effectiveness rates for the 5–17 and 0–4 year age-groups. Because of concerns that these corrections might, in and of themselves, inadvertently introduce additional biases, we elected to present the uncorrected values in the main body of the manuscript. Further discussion of the potential implications of the bias studies is provided in the S1 Text material accompanying the manuscript.

In considering possible reasons for the non-negligible summer ILI rates: Alachua County has had an aggressive SLIV program since the fall of 2009, with overall immunization rates among schoolchildren consistently above 40%. It may be that maintenance of consistently high levels of immunity in a community among the age-group associated with elevated influenza transmission, *i.e.,* school-age children and young adults, over a period of several years resulted in longer periods of transmission, but at lower rates. Further studies are warranted of the physiologic, virologic, sociologic, and/or epidemiologic mechanisms underlying this reduction, given that circulation of influenza is thought to be negligible during such non-epidemic periods.

## Conclusions

Our results show that immunization of school-age children with influenza vaccination protects them from ILI-associated outpatient EC visits, and also protects the very young, one of the most vulnerable age-groups for adverse morbidity and mortality outcomes. [3] This study highlights the value of SLIV programs for increasing influenza immunization coverage rates by complementing the traditional delivery strategy that depends upon medical offices and pharmacies. Through the SLIV program, we have observed increase vaccination uptake among minorities and lower socioeconomic groups that may not normally get vaccinated. [5] We hope this study will promote dialogue among the members of the research community and the general public regarding the effectiveness of the SLIV approach for reducing the public health burden attributable to influenza, particularly as wider implementation of routine SLIV programs is being considered in the USA and abroad (for example, the United Kingdom [7]). Our work provides a compelling argument for further community-based research into the nature and extent of the impact of SLIV programs.

## Supporting Information

S1 Figure **Among the 0 to 4 year-old residents of Alachua County (gray bars), the rest of Region 3 (dashed line), and Florida (excluding Alachua County, solid line), the weekly rates of outpatient visits (per 100,000 residents) to sentinel emergency room and urgent care departments for chief complaints associated with influenza-like illness.** The epidemic periods are denoted by thick horizontal lines located at the base of the plot.
(TIF)

S2 Figure **Among the 5 to 17 year-old residents of Alachua County (gray bars), the rest of Region 3 (dashed line), and Florida (excluding Alachua County, solid line), the weekly rates of outpatient visits (per 100,000 residents) to sentinel emergency room and urgent care departments for chief complaints associated with influenza-like illness.** The epidemic periods are denoted by thick horizontal lines located at the base of the plot.
(TIF)

S1 Text **Sensitivity analysis for ascertainment bias in the estimation of the effectiveness of the school-located influenza vaccination program in Alachua County, Florida, from 2011–2013.**
(DOCX)
