# Supplementary figures and images for: School-Located Influenza Vaccination Reduces Community Risk for Influenza and Influenza-Like Illness Emergency Care Visits
Source: PLoS One. 2014 Dec 9;9(12):e114479. doi: 10.1371/journal.pone.0114479 (PMC4260868; doi:10.1371/journal.pone.0114479)

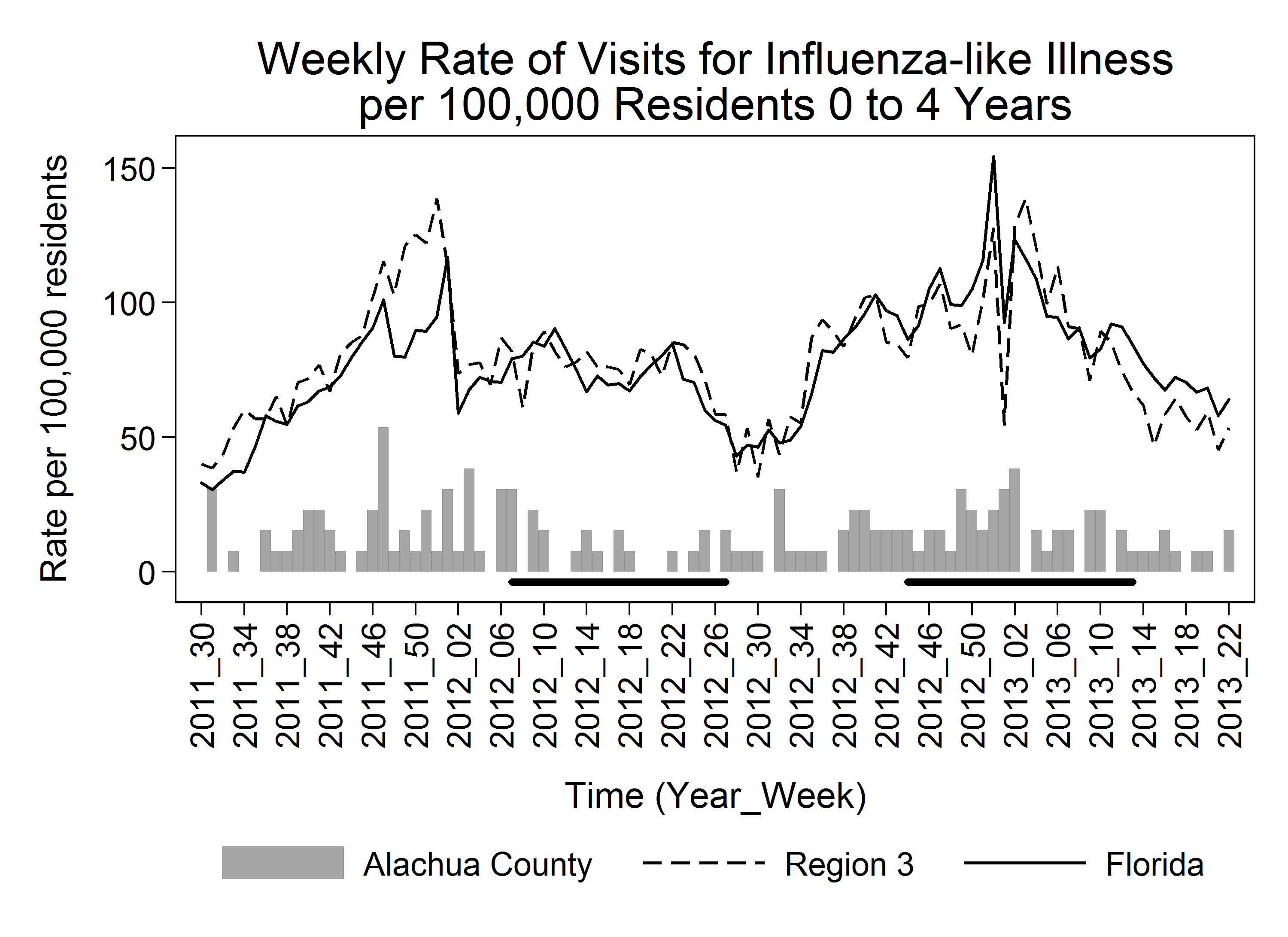

Supplement: S1 Figure — Among the 0 to 4 year-old residents of Alachua County (gray bars), the rest of Region 3 (dashed line), and Florida (excluding Alachua County, solid line), the weekly rates of outpatient visits (per 100,000 residents) to sentinel emergency room and urgent care departments for chief complaints associated with influenza-like illness. The epidemic periods are denoted by thick horizontal lines located at the base of the plot. (TIF) [file pone.0114479.s001.tif]

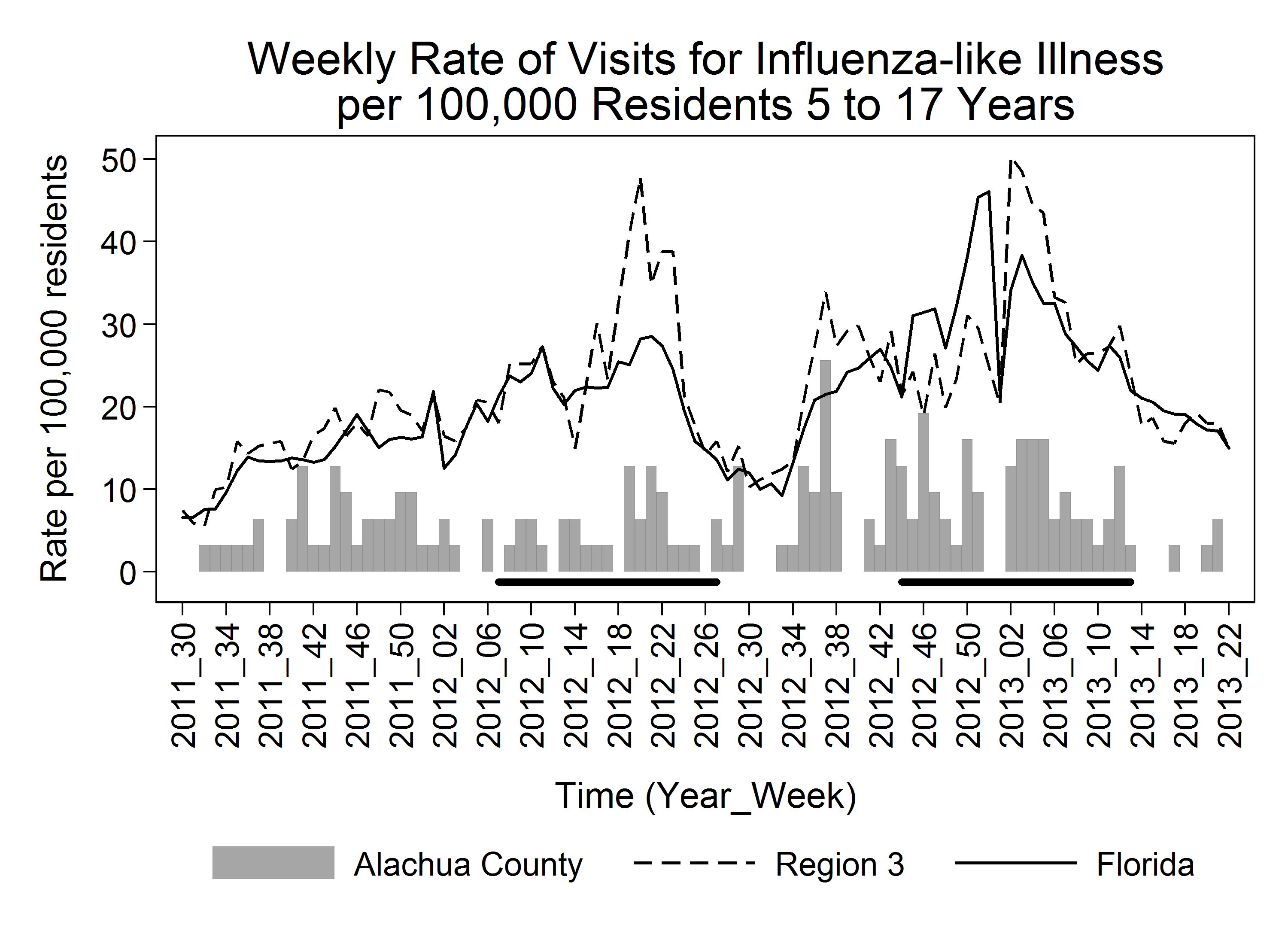

Supplement: S2 Figure — Among the 5 to 17 year-old residents of Alachua County (gray bars), the rest of Region 3 (dashed line), and Florida (excluding Alachua County, solid line), the weekly rates of outpatient visits (per 100,000 residents) to sentinel emergency room and urgent care departments for chief complaints associated with influenza-like illness. The epidemic periods are denoted by thick horizontal lines located at the base of the plot. (TIF) [file pone.0114479.s002.tif]
