# Supplementary material for: School-Located Influenza Vaccination Reduces Community Risk for Influenza and Influenza-Like Illness Emergency Care Visits
Source: PLoS One. 2014 Dec 9;9(12):e114479. doi: 10.1371/journal.pone.0114479 (PMC4260868; doi:10.1371/journal.pone.0114479)
Supplement: S1 Text — Sensitivity analysis for ascertainment bias in the estimation of the effectiveness of the school-located influenza vaccination program in Alachua County, Florida, from 2011–2013. (DOCX) [file pone.0114479.s003.docx]

**SUPPLEMENTARY MATERIAL**

**Manuscript** **Title:** School-Located Influenza Vaccination Reduces Community Risk for Influenza and Influenza-like Illness Emergency Care Visits

**Supplementary Appendix A. Sensitivity analysis for ascertainment bias in the estimation of the effectiveness of the school-located influenza vaccination program in Alachua County, Florida, from 2011-2013.**

Information on the frequency of respiratory illness captured by syndromic surveillance systems is routinely used to estimate the effectiveness of influenza vaccination.^1-9^ These types of studies are typically inexpensive and efficient to conduct using existing information about the frequency of symptomatic influenza in a study population. Despite the advantages of this approach for estimating influenza vaccine effectiveness, there is a growing body of evidence that observational studies of influenza vaccine effectiveness are prone to confounding and bias.^10-14^ Ecologic studies using data from routine syndromic surveillance systems are particularly prone to potential bias resulting from differential ascertainment and/or misclassification of symptomatic influenza cases between comparison groups.

One method for investigating and/or accounting for potential bias and confounding in these types of studies is through the use of negative control outcomes. Among elderly members of a health maintenance organization, Jackson *et al.*^11^ employed negative controls to investigate potential confounding of estimated influenza vaccination effects by differences between vaccinated and unvaccinated individuals in their general state of health. Lipsitch, Tchetgen Tchetgen, and Cohen^15^ subsequently specified a conceptual framework for the use of negative controls conditions in the epidemiologic studies, using the Jackson *et al.*^11^ study as an illustrative example.

The current ecologic study estimated the effectiveness of a novel school-located influenza vaccination (SLIV) program in Alachua County, Florida. This SLIV has achieved relatively high influenza vaccination coverage rates among school-age children in the county since 2009.^16^ The current study estimated the SLIV’s effectiveness toward reducing the attack rate for outpatient visits to sentinel emergency departments and urgent care facilities where influenza-like illness was the chief complaint (primary outcome: ILI-associated outpatient visits to sentinel facilities). The effectiveness of this program at reducing the risk of ILI-associated outpatient visits in the entire community and the effectiveness by age-group are estimated for Alachua County relative to two comparison areas: the 12 surrounding counties (Region 3) and all non-Alachua counties of Florida (Florida). The SLIV program’s effectiveness was high among children under-18 years-old and decreased steadily with increasing age.

Through a sensitivity analysis, this supplementary appendix explores and accounts for potential ascertainment bias in the unadjusted estimates of the effectiveness of the Alachua County SLIV program that are presented in the main manuscript. Differential ascertainment of ILI cases between the surveillance sites in Alachua and the comparison areas (Region 3 and rest of Florida) is thought to be a significant source of potential ascertainment bias in the unadjusted estimates of the SLIV program’s effectiveness. Unfortunately, insufficient information was available to explicitly characterize the set of processes leading to the ascertainment of a case. Therefore, the following additional information collected by the same sentinel surveillance system are used to explore and account for potential ascertainment bias: the weekly rate for all outpatient visits to sentinel providers and the attack rates for outpatient visits associated with each of three negative control outcomes during the epidemic periods.

**A.1. Methods**

**Primary and negative control outcomes.** Negative control outcomes collected during the epidemic season provide a means of investigating potential bias due to time-dependent differential ascertainment. The ideal negative control outcome would have the same probability of being detected by the surveillance system during the epidemic period (*i.e.*, identical propensity for seeking healthcare at a sentinel facility and the same probability of being correctly identified in the electronic reporting system), but would not be associated with the exposure or a cause of the primary outcome.^15^

Three control conditions are considered for the current study. All three conditions are captured in the same manner as ILI by the same ESSENCE surveillance system. See Supplementary Appendix B for the surveillance case definitions for the primary and negative control outcomes. Outpatient visits to sentinel facilities for gastrointestinal complaints and other respiratory diseases (negative control conditions 1 and 2) are expected to be fairly compatible to ILI with regard to the probability of being detected. These two negative control conditions suffer from one potential drawback; they are likely to be moderately associated in this dataset with the primary exposure (SLIV program) through misclassification of a proportion of true ILI-associated outpatient visits as either of these negative control conditions. This proposed association between the study exposure and the negative control outcomes 1 and 2 would only exist if the SLIV program actually affects the risk of ILI-associated outpatient visits in Alachua County. The final negative control outcome, outpatient visits due to a physical injury (negative control outcome 3), is unlikely to be associated with the primary exposure, but may exhibit a different set of causal factors from ILI-associated visits (for example, a higher propensity to seek outpatient care for an injury relative to ILI).

SLIV effectiveness estimates for an epidemic season are separately biased-corrected for each negative control outcome and corrected for the average effect of the three negative control outcomes. Bias-correction for each negative control outcome was estimated as the exponentiation of the difference between the relative risks for ILI and for the negative control outcome on the natural logarithm scale. Simultaneous bias-correction for all three negative control outcomes was estimated in the same manner using the geometric mean of the relative risks for the three negative control outcomes.

**Statistical methods.** The data consists of counts $i_{o,a,c,t}$ of outpatient visits to sentinel emergency department and urgent care facilities for each outcome $o$ (ILI=influenza-like illness; Resp=Other respiratory illness; GI=gastrointestinal illness; Injury=physical injury), age-group $a$ in years (0-4; 5-17; 18-44; 45-64; 65plus), geographic area $c$ (A=Alachua; R=Region 3; F=Rest of Florida), and week $t$ (CDC week and calendar year). Weekly counts $k_{a,c,t}$ of all outpatient visits for any reason are similarly indexed by age-group $a$, geographic area $c$, and week $t$. The number of residents from the US 2010 Census,^17^ $m_{a,c}$, is indexed by age-group $a$ and geographic area $c$. The unstandardized attack rate for outcome $o$ during the epidemic period $p$ (1=2011-2012; 2=2012-2013) among residents of geographic area $c$ in age-group $a$ is estimated by

$${AR}_{o,p,a,c,S-}=\frac{\sum_{t\in p} i_{o,a,c,t}}{m_{a,c}}$$

, where $\text{S-}$ represents the unstandardized state for the process $x$ of standardization for the rate of outpatient visits of any type and $t\in p$ represents the set of weeks comprising the epidemic period $p$.

*Standardization for the rate of outpatient visits of any type*. The weekly rate of outpatient visits to sentinel emergency departments and urgent care facilities for any reason differs between Alachua County and the comparison areas. This difference is a potential source of bias with regard to the ascertainment of ILI among community members. To explore the effects of and account for any ascertainment bias from this source, the weekly rate for the primary outcome in each of the comparison areas was standardized to the overall visit rates for the same week in Alachua County. The visit-rate standardized attack rate for outcome $o$, epidemic period $p$, age-group $a$, and geographic area $c$ is estimated by

$${AR}_{o,p,a,c,S+}=\frac{\sum_{t\in p} \left( \frac{i_{o,a,c,t}}{k_{a,c,t}}*\frac{\frac{k_{a,c,t}}{m_{a,c}}}{\frac{k_{a,A,t}}{m_{a,A}}} \right)}{m_{a,c}}$$

*Unadjusted and visit-rate standardized relative risk*. The ratio of the attack rates among residents of Alachua County versus each comparison area is the primary focus of this sensitivity analysis, with SLIV effectiveness estimated as 1 minus this relative risk.^18^ To explore the potential effects of ascertainment bias on SLIV effectiveness, a set of relative risks, ${RR}_{o,p,a,c,x}^{1}$, are estimated for strata indexed by outcome $o$, epidemic period $p$, age-group $a$, non-Alachua geographic comparison area $c$, and visit-rate standardization status $x$ by

$${RR}_{o,p,a,c\neq A,x}^{1}=\frac{{AR}_{o,p,a,A,x}}{{AR}_{o,p,a,c\neq A,x}}\text{.}$$

*Bias correction of the relative risk for ILI using negative control outcomes*. Both unadjusted and bias-corrected estimates for the relative risk of ILI-associated outpatient visits to sentinel facilities, ${RR}_{a,p,c,x,z}^{2}$, are indexed by age-group $a$, epidemic period $p$, non-Alachua geographic comparison area $c$, visit-standardization status $x$, and by the status $z$, indicating the negative control outcome used for bias correction (None; Resp=Other respiratory illness; GI=gastrointestinal illness; Injury=physical injury; All = geometric mean of Resp, GI, and Injury). This relative risk is estimated by

$${RR}_{a,c\neq A,x,z}^{2}=e^{\left[ \ln\left( {RR}_{ILI,a,p,c\neq A,x}^{1} \right)-\ln\left( g(.) \right) \right]}$$

, where

$$g\left( . \right)=\left\{ \begin{aligned} 1 z=None \\ {RR}_{z,p,a,c\neq A,x}^{1} z\neq None and z\neq All \\ e^{\left[ \frac{\ln\left( {RR}_{Resp,p,a,c\neq A,x}^{1} \right)+\ln\left( {RR}_{GI,p,a,c\neq A,x}^{1} \right)+\ln\left( {RR}_{Injury,p,a,c\neq A,x}^{1} \right)}{3} \right]} z=All \end{aligned} \right.\text{.}$$

Ninety-five percent confidence intervals for bias-corrected relative risk estimates are estimated using a parametric bootstrapping procedure^19^ that resamples with replacement the number of cases of each outcome within strata defined by epidemic period and geographic area. Two hundred samples are drawn from a binomial probability distribution with mean equal to the attack rate observed within each geographic area during an epidemic season.

**A.2. Results**

The weekly rates of the outpatient visits per 100,000 residents for each of the study outcomes (primary and negative controls) and for all outpatient visits to a sentinel facility vary between Alachua County and the comparison areas. Within each area, these rates also vary over time and between age-groups. Outpatient visit rates demonstrate distinct seasonality for ILI and other respiratory illness, but not for the other outcomes or for all outpatient visits. Among individuals under 18 years-olds (figure S2 and S3), the unadjusted weekly attack rates for ILI-associated outpatient visits are distinctly lower among Alachua County residents relative to both comparison areas, and this difference is less pronounced for the other outcomes and for all outpatient visits. Among young adults 18-44 years-of-age, which includes Alachua County’s large university student population, there are distinctly lower weekly rates in Alachua County relative to both comparison areas for each of the study outcomes and for all outpatient visits. For adults over 44 years-old, differences between Alachua County and the comparison areas in the rates of each of the study outcome and all outpatient visits are not very pronounced. When considering the rates of the study outcomes and of all outpatient visits among individuals of any age, the temporal and spatial patterns are similar to those observed among individuals under 18 years of age.

The visit-rate standardization procedure affected the value of the relative risk estimate. Since the direction and the magnitude of the shift in the value of the relative risk are consistent for a given age-group within a comparison area, all bias-corrected relative risk and effectiveness estimates will be visit-rate standardized.

The visit-rate standardized unadjusted relative risk estimates for each of the primary and negative control outcomes by age-group and comparison area. Among individuals under 18 years-of-age, the visit-rate standardized relative risk for ILI remains consistently protective in both epidemic periods. This trend is observed, to a lesser extent, among older age-groups. Protective visit-rate standardized relative risk estimates for the other respiratory and gastrointestinal illness outcomes are also observed for most time periods among individuals 18 years and older. The visit-rate standardized relative risk estimates for injury range around the null value of 1.0. Table 3 & Table 4 (main text) present the effects of the bias correction methods on the estimated effectiveness of the SLIV program by age-group and epidemic period. Bias correction had little impact on the interpretation of the point estimate for the overall effectiveness of the SLIV program among 5 to 17 year-olds and the indirect effectiveness of the SLIV program among children under 5 years-old and adults over 44 years-of-age.

**A.3 Discussion**

Our results demonstrate that there is little evidence of ascertainment bias during the epidemic season. The indirect effectiveness estimates among children under 5 years-old and the overall effectiveness among school-age children (5-17 years) are slightly lower in value when corrected for bias. In contrast, after correction for potential observation bias, the estimated effectiveness tends to increase slightly among individuals 18 years and older.

**Supplementary Appendix B. Surveillance case definitions for study outcomes**

The ESSENCE system queries key terms in the description of the chief complaint associated with an outpatient visit to a sentinel emergency department or urgent care facility. Using a set of algorithms, the results of the key term search are used to automatically assign each outpatient visits to one of 12 syndrome categories. Each of the following sections lists the key terms that are used to define the primary and negative control outcomes for the current study.

**B.1. Influenza and Influenza-like Illness (ILI) syndrome category:**

Includes:

- “influenza”
- “fever and cough”
- “fever and sore throat”

Excludes all non-ILI fevers.

**B.2. Other Respiratory Illness syndrome category:**

Includes at least one of the following sub syndromes:

- “acute bronchitis”
- “congestion chest”
- “cough”
- “difficulty breathing”
- “hemoptysis”
- “laryngitis”
- “lower respiratory infection”
- “congest nasal”
- “otitis media”
- “pneumonia”
- “shortness of breath”
- “sore throat”
- “upper respiratory infection”
- “wheezing”
- “acute respiratory distress”

**B.3. Gastrointestinal Illness (GI) syndrome category**

Includes at least one of the following sub syndromes:

- “abdominal pain”
- “bloating”
- “gastroenteritis”
- “GI bleeding”
- “loss of appetite”
- “nausea”
- “vomiting”
- “diarrhea”
- “food poisoning”

**B.4. Physical Injury syndrome category**

Includes at least one of the following sub syndromes:

“bite or sting”

“cut or pierce”

“drowning or submersion”

“electrocution”

“excessive heat”

“fall”

“fire burn explosives”

“motor vehicle”

“occupational”

“overexertion”

“poisoning”

“struck by”,

“tools or machinery”

“firearm”

“non-motor vehicle”

“suffocation”

“assault”

“foreign body”

“suicide or self-inflected”

“water craft”

“sports or exercise related”

**Supplementary Appendix C. Bibliographic References**

1. Centers for Disease C, Prevention. Assessment of the effectiveness of the 2003-04 influenza vaccine among children and adults--Colorado, 2003. *MMWR Morb Mortal Wkly Rep* 2004; **53**(31): 707-10.

2. Centers for Disease C, Prevention. Effectiveness of 2008-09 trivalent influenza vaccine against 2009 pandemic influenza A (H1N1) - United States, May-June 2009. *MMWR Morb Mortal Wkly Rep* 2009; **58**(44): 1241-5.

3. Centers for Disease C, Prevention. Interim results: state-specific seasonal influenza vaccination coverage - United States, August 2009-January 2010. *MMWR Morb Mortal Wkly Rep* 2010; **59**(16): 477-84.

4. Centers for Disease C, Prevention. Estimated influenza illnesses and hospitalizations averted by influenza vaccination - United States, 2012-13 influenza season. *MMWR Morb Mortal Wkly Rep* 2013; **62**(49): 997-1000.

5. Centers for Disease C, Prevention. Interim adjusted estimates of seasonal influenza vaccine effectiveness - United States, February 2013. *MMWR Morb Mortal Wkly Rep* 2013; **62**(7): 119-23.

6. Centers for Disease C, Prevention. Early estimates of seasonal influenza vaccine effectiveness--United States, January 2013. *MMWR Morb Mortal Wkly Rep* 2013; **62**(2): 32-5.

7. de Oliveira Jde F, Boing AF, Waldman EA, Antunes JL. Ecological study on mortality from influenza and pneumonia before and after influenza vaccination in the Northeast and South of Brazil. *Cadernos de saude publica* 2013; **29**(12): 2535-45.

8. Ridenhour BJ, Campitelli MA, Kwong JC, et al. Effectiveness of inactivated influenza vaccines in preventing influenza-associated deaths and hospitalizations among Ontario residents aged >/= 65 years: estimates with generalized linear models accounting for healthy vaccinee effects. *PloS one* 2013; **8**(10): e76318.

9. Thompson MG, Shay DK, Zhou H, et al. Estimates of deaths associated with seasonal influenza --- United States, 1976-2007. *MMWR Morb Mortal Wkly Rep* 2010; **59**(33): 1057-62.

10. Ferdinands JM, Gargiullo P, Haber M, Moore M, Belongia EA, Shay DK. Inactivated influenza vaccines for prevention of community-acquired pneumonia: the limits of using nonspecific outcomes in vaccine effectiveness studies. *Epidemiology (Cambridge, Mass)* 2013; **24**(4): 530-7.

11. Jackson LA, Jackson ML, Nelson JC, Neuzil KM, Weiss NS. Evidence of bias in estimates of influenza vaccine effectiveness in seniors. *Int J Epidemiol* 2006; **35**(2): 337-44.

12. Jackson ML, Yu O, Nelson JC, et al. Further evidence for bias in observational studies of influenza vaccine effectiveness: the 2009 influenza A(H1N1) pandemic. *Am J Epidemiol* 2013; **178**(8): 1327-36.

13. Nelson JC, Jackson ML, Weiss NS, Jackson LA. New strategies are needed to improve the accuracy of influenza vaccine effectiveness estimates among seniors. *Journal of clinical epidemiology* 2009; **62**(7): 687-94.

14. Nelson JC, Marsh T, Lumley T, et al. Validation sampling can reduce bias in health care database studies: an illustration using influenza vaccination effectiveness. *Journal of clinical epidemiology* 2013; **66**(8 Suppl): S110-21.

15. Lipsitch M, Tchetgen Tchetgen E, Cohen T. Negative controls: a tool for detecting confounding and bias in observational studies. *Epidemiology (Cambridge, Mass)* 2010; **21**(3): 383-8.

16. Tran CH, McElrath J, Hughes P, et al. Implementing a community-supported school-based influenza immunization program. *Biosecurity and bioterrorism : biodefense strategy, practice, and science* 2010; **8**(4): 331-41.

17. U.S. Census Bureau, 2010 Census of Population and Housing, Demographic Profile Summary File 1. http://www2.census.gov/census_2010/04-Summary_File_1/Florida/fl2010.sf1.zip (Accessed September 2013).

18. Halloran ME, Longini IM, Jr., Struchiner CJ. Design and Analysis of Vaccine Studies. 1 ed. New York: Springer; 2010.

19. Efron B, Tibshirani RJ. An Introduction to the Bootstrap. Boca Raton, Florida: Chapman & Hall/CRC; 1994.
